# Impacts of event-specific air quality improvements on total hospital admissions and reduced systemic inflammation in COPD patients

**DOI:** 10.1371/journal.pone.0208687

**Published:** 2019-03-20

**Authors:** Zili Zhang, Jian Wang, Fei Liu, Liang Yuan, Jili Yuan, Lianghua Chen, Nanshan Zhong, Wenju Lu

**Affiliations:** 1 State Key Laboratory of Respiratory Disease, Guangzhou Institute of Respiratory Disease, The First Affiliated Hospital, Guangzhou Medical University, Guangzhou, Guangdong, P.R. China; 2 Division of Translational and Regenerative Medicine, Department of Medicine, The University of Arizona, Tucson, AZ, United States of America; 3 Department of Pediatrics, The First Affiliated Hospital, Guangzhou Medical University, Guangzhou, Guangdong, P.R. China; 4 Department of Laboratory Medicine, The First Affiliated Hospital, Guangzhou Medical University, Guangzhou, Guangdong, P.R. China; University of Notre Dame Australia, AUSTRALIA

## Abstract

There is limited evidence linking the impacts of reduced air pollution on hospital admissions. The potential biological mechanisms are still not completely understood. This study examined the effects of mitigated ambient pollution on hospital admissions and inflammatory biomarker levels in chronic obstructive pulmonary disease (COPD) COPD patients. Daily hospital admissions were compared over 51 days associated with the Asian Games period (Nov 1-Dec 21, 2010) with the identical calendar dates of baseline years (2004–2009 and 2011–2013). A three-year cohort study was conducted with 36 COPD patient participants. The daily particulate matter (PM_10_) decreased from 65.86 μg/m^3^ during the baseline period to 62.63 μg/m^3^ during the Asian Games period; the daily NO_2_ level decreased from 51.33 μg/m^3^ to 42.63 μg/m^3^. Between the baseline period and the Asian Games, daily hospital admissions from non-accidental diseases decreased from 116 to 93, respectively; respiratory diseases decreased from 20 to 17, respectively; and cardiovascular diseases decreased from 11 to 9 during the Asian Games period, respectively. No statistically significant reductions were seen in the remaining months of 2010 in Guangzhou, during the the Asian Games period in the control city, and two other control diseases. Furthermore, we identified significant improvement in CRP and fibrinogen by -20.4% and -15.4% from a pre-Asian game period to a during-Asian game period, respectively. For CRP, we found significant increases in NO_2_ at lag1-3 days after-Asian game period and significant increases in PM_10_ at lag1-2 days. Similar effects were also seen with fibrinogen. This discovery provides support for efforts to diminish air pollution and improve public health through human air pollutants intervention. Improved air pollution during the 2010 Asian games was correlated with decreases in biomarkers associated with systemic inflammation in COPD patient participants.

## Introduction

Over the past decades, studies have linked short-term high concentrations of air pollutants to hospital admissions, most mainly due to respiratory and cardiovascular diseases [1, 2]. Evidences for air pollution mitigation is limited by failing to mention that prior study has shown an association between air pollution reduction and hospitalizations [3–5]. These linkages highlight the need to develop effective environmental policies or air quality legislation to improve air pollution; however, questions remain about whether improved air pollution resulting from air pollution control measures lead to public-health benefits [6, 7]. Fortunately, large-scale, multi-sport events and emissions control from industries have provided new research opportunities. Previous findings (such as the Beijing Olympic Games in 2008) have supported efforts to diminish air pollution and improve public health by controlling industrial emissions and limiting transportation [8]. Efforts are still needed to explore the health effects of air quality that has been improved through human air pollutants intervention.

Automobile exhaust, industry emissions, and power generation plants are primary sources of air pollution. In the past three decades, worldwide automobile use has significantly influenced relative amounts of fine particulate matter (PM_2.5_), nitrogen oxides (NOx), and carbon monoxide (CO) emitted from vehicles, compared to emissions from non-vehicle sources. However, epidemiologic evidence about the association between ambient pollution mitigation and hospital admissions for total, cardiovascular, and respiratory diseases has not been elucidated completely, especitally in the Asian region. As a condition for hosting the 16th Asian Games, as well as the Asian Para Games in Guangzhou, the municipal government invested unprecedented effort to improve air quality. The air pollution improvements and favorable weather circumstances led to an unusually low level of pollutants. This created a unique opportunity to evaluate the acute health response to the mitigated pollution during the Asian Games period.

However, the underlying biological mechanisms have not been well explained. Systemic inflammation is an important feature of chronic obstructive pulmonary disease (COPD) [9]. The inflammation is characterized by increased circulatory inflammatory biomarker levels, such as CRP, fibrinogen, and interleukin (IL)-6, IL-8 [10]. In addition, the origins of inflammation in COPD patients are not known. Although an inflammatory response resulting from ambient air pollution exposure may significantly influence the production of inflammatory markers, there are limited studies about the effects of air pollution on COPD. Existing studies about the relationship between air pollution and inflammatory biomarkers have resulted in conflicting conclusions [11]. This study explored the effects of air pollution exposure on the CRP and fibrinogen of systemic inflammation in 36 clinically stable COPD patients participating in a three-year cohort study (2009–2011). Data were based on the Guangzhou Institute of Respiratory Disease (GIRD) COPD Biobank study [12]. This is the first study we know of that has capitalized on air pollution intervention opportunities to assess mechanistic biomarkers in COPD.

Therefore, we aimed to examine whether there were any decreases in short-term hospital admissions resulting from mitigated air pollution. Then, we further wanted to see if CRP and fibrinogen decreases with reduction in air pollution in COPD patients.

## Materials and methods

### Study area and population

The study area and polulation has been described in detail elsewhere[9, 13]. This study was conducted in Guangzhou, Guangdong, PR, China. Guangzhou includes nine different districts and many counties. In 2000, the population of Guangzhou was approximately 13.2 million, representing 1% of China’s total population. The target population included permanent inhabitants in the Haizhu District (90.4 km^2^), which is one of the districts in Guangzhou, with a population of approximately 1.55 million in 2015. Two continuous state-controlled air pollution-monitoring sites were situated in the area. Ambient air pollution in Haizhu district is mainly caused by emissions from automobile exhaust. The predominant air pollutants include NO_2_ (nitrogen dioxide), PM_10_ (particulate matter, a diameter measuring less than 10μm), and SO_2_ (sulfur dioxide).

### Daily hospital admissions data

Data on hospital admissions have also been described previously elsewhere [13]. In brief, the data were collected from the Emergency department of the Haizhu District Health Insurance Bureau, managed by a government agency in China. For this study, we first checked emergency visit data from the emergency department; if patients were hospitalized, hospital admissions data were attained. The International Classification of Disease 10th Revision (ICD-10) was used, and total non-accidental and cause-specific hospital admissions, excluding accidents and injuries (A00-R99) were estimated for the study. This included respiratory illnesses (J00-J98, except J00X02-J00X04), cardiovascular illnesses (I00-I99), digestive illnesses (K00-K99), and urogenital illnesses (N00-N99). The study characteristics have been described in detail elsewhere, including ICD-10 diagnosis codes, disease categories, participant ages and sex [9].

### Clinical data

As described in the previous section, we used a panel design for this study, where each participant was required to finish 6 clinical visits: 2 visits before the Asian Games period (Nov 1-Dec 21, 2009), 2 during the Asian Games period (Nov 1-Dec 21, 2010), and 2 after the Asian games (Nov 1-Dec 21, 2011). At the beginning, there were 41 study participants. Five participants who completed only 1 or 2 visits were excluded from the analysis. Data for the 36 remaining participants who attended all 6 visits were used in all data analyses. The included subjects from the GIRD COPD Biobank Project established by Guangzhou Institute of Respiratory Disease and State Key Laboratory of Respiratory Disease, the First Affiliated Hospital Guangzhou Medical University, Haizhu District, Guangzhou, Guangdong, PR, China. Briefly, the study team recruited COPD patients older than 40 years with chronic respiratory symptoms during their first hospital admission resulting from an exacerbation. Blood samples were measured using immunonephelometry for fibrinogen and CRP (Dade Behring Marburg GmbH, Marburg, Germany). All tests were conducted in the translational medicine laboratory of the First Affiliated Hospital Guangzhou Medical University. The study protocol was approved by the Ethics Committee of the First Affiliated Hospital. Informed consent was obtained from all patients before participating in the study.

### Air pollution and meteorological data

**Fig 1:**
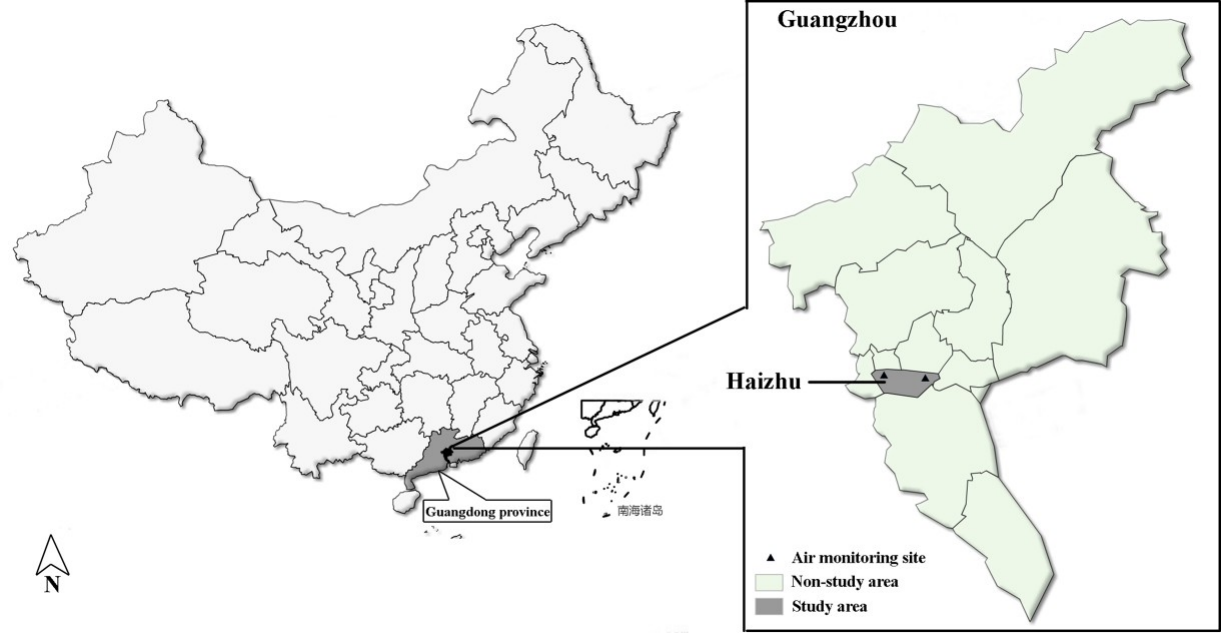
District map of Haizhu District, Guangzhou, China, with two air monitoring station locations.

Ambient pollution data were provided by the Guangzhou Municipal Environmental Protection Monitoring Center. Daily pollutant concentrations in outdoor air were calculated as mean values collected from the two monitoring sites in Haizhu district (Fig 1). The monitoring sites were automated and provided daily readings of NO_2_, PM_10_, and SO_2_. The color differential optical absorption spectroscopy (DOAS) method was used to assess NO_2_ and SO_2_, and β-ray absorption method was used to determine PM_10_. In addition, daily mean temperature and relative humidity were also collected, to adjust for the impact of weather on hospital admissions. The entire monitoring standard is consistent with the International World Meteorological Organization standard (https://library.wmo.int/doc_num.php?explnum_id=4065). The air pollutants mitigated policy, encompassing the Asian Games and Asian Para Games (Nov 1-Dec 21), mainly included transportation restrictions and emissions control from industries. For example, vehicles were only allowed to be driven on alternate days under an even-odd license plate system; heavy duty trucks were forbidden on roads; and industrial factories must be closed [14].

### Statistical analysis

Daily air pollution concentration and meteorological variables during the Asian Games period were analyzed and compared to the baseline period. The student’s *t* test was used to determine whether daily air pollutant concentrations and meteorological variables differed significantly between the two study periods. Repeated measures analysis of variance was used to describe the baseline characteristics for the cohort of 36 COPD patients.The significance level was defined as *P* < 0.0083 (To control the family-wise type I error rate at a 0.05 level, a Bonferroni correction was applied. With 6 between-period comparisons (2 biomarkers by 3 between-period changes), each individual 2-sided test was considered statistically significant relative to a 0.0083 significance level). To estimate whether any decline in hospital admissions was attributable to lower pollutant concentrations during the 51 days of the 2010 Asian Games (Nov 1-Dec 21), we calculated daily hospital admissions counts during the Asian Games period, and during the same time period for the six years before (2004–2009) and the three years after (2011–2013). These years before and after the Asian Games are defined as the baseline period. The relationships between air control measures and daily hospital admissions were evaluated using a time series Poisson Regression Generalized Additive Model (GAM). The univariate and adjusted RR (with 95% confidence interval (95%CI)) of hospital admissions from total non-accidental, respiratory, and cardiovascular diseases was calculated to compare the the Asian Games period and the baseline period. Different potential confounding factors were adjusted in the multivariate model. Seasonality and time trends were not measured in the multivariate models, because the intervention period was a very short period only in the winter season [15]. The model is expressed as:

$$Log(u_{t})=\alpha +Game+DOW+Holiday+COVs$$

In this expression, u_t_ represents the daily hospital admissions counts; Game was defined as a binary variable that was 1 for the intervention days and 0 for the baseline days; DOW referred to day of week; Holiday was a binary variable indicating public holidays; and COVs were the other potential factors, such as daily mean temperature and relative humidity.

In addition, we estimated each biomarker’s change from the baseline period to the Asian Games period, and from the Asian Games period to the baseline period. The biomarkers served as the dependent variable and the time period served as the independent in Mixed-Linear Models (MLM). Bonferroni corrections were used to control the family-wise type I error rate at 0.05. We assessed the change in biomarker concentration associated with a 10 μg/m^3^ increase in pollutant concentration using MLM. Then, it was used to estimate the change in biomarker levels related to each 10 μg/m^3^ increase in pollutant concentrations during the day before the clinic visit (lag 0), and the previous 6 days (lag 1–6). The mean percentage change and its 95%CIs were calculated for the biomarker associated with each 10 μg/m^3^ pollutant increase. Spearman was used to the correlation analysis between Meteorology and air pollutants.

All analyses were performed using the mgcv, gls, and lme package in R-project (version 2.14.1).

## Results

Table 1 shows the air pollution, daily hospital admissions, and meteorological factors for the 2010 Asian Games and for the same period during 2004–2009 and 2011–2014 in Guangzhou (S1 Fig). Daily total non-accidental hospital admissions decreased from 116 per day during the baseline to 93 during the Asian Games period, which was a 20.05% overall decrease **(***P* = 0.011, Table 1**).** Daily hospital admissions on cardiovascular disease and respiratory disease decreased from 11 to 9 and from 20 to 17, respectively. This corresponded to an overall 19.25% (*P* = 0.021) and 14.95% (*P* = 0.022) decrease in cardiovascular disease and respiratory disease, respectively.

**Table 1:** Comparison of daily hospital admissions, air pollution and meteorological conditions between the intervention period and the baseline period in Guangzhou.

Variables	Mean(SD)		Change%	*P*
	Baseline period ^b^	Intervention period ^c^		
**Hospital admission**				
**Non-accidental**	116(62)	93(52) ^a^	-20.05	**0.011**
**Cardiovascular**	11(6)	9(4) ^a^	-19.25	**0.021**
**Respiratory**	20(9)	17(7) ^a^	-14.95	**0.022**
**Pollutant**				
**PM**_**10**_ **(**μ**g/m**^**3**^**)**	65.86(26.22)	62.63(14.39)	-4.90	0.388
**NO**_**2**_ **(**μ**g/m**^**3**^**)**	51.33(27.63)	42.63(14.33) ^a^	-16.95	**0.027**
**SO**_**2**_ **(**μ**g/m**^**3**^**)**	30.30(16.71)	30.65(15.09)	1.16	0.886
**Meteorological**				
**Temperature (**°**C)**	19.18(4.27)	19.37(3.54)	0.99	0.760
**Relative humidity (%)**	59.18(15.75)	57.73(12.08)	-2.45	0.525

^a^ *P* < 0.05 in t test

^b^ Baseline period: November 1-December 21 from 2004 to 2013, except 2010; a negative sign represent a decrease between baseline and intervention period.

^c^ Intervention period: November 1-December 21 in 2010.

**Fig 2:**
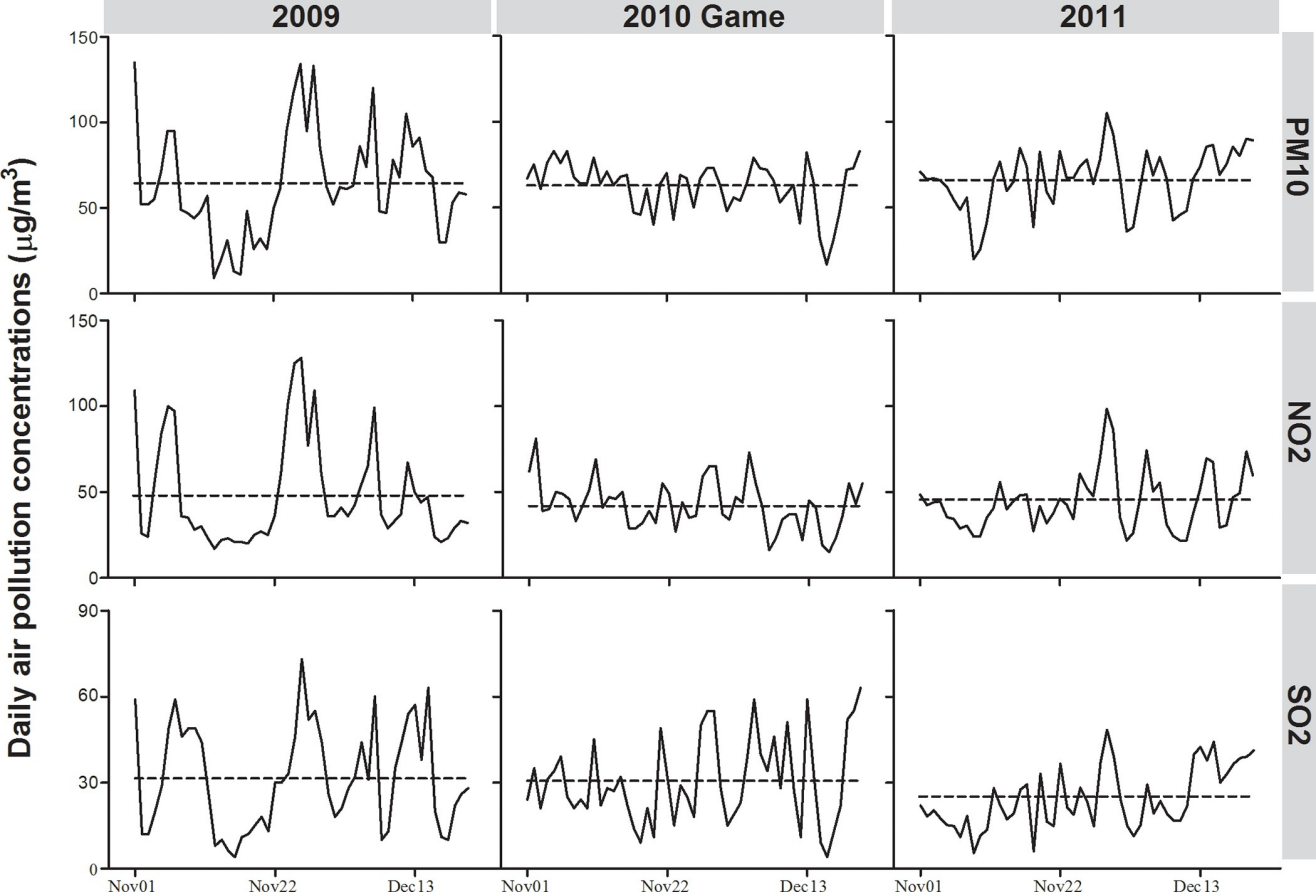
Time series of daily air pollution concentrations during the 2010 Asian Games period and during the baseline years in Guangzhou.

Table 2 displays the RR by hospital admission cause, age group, and sex. Confounding factor were adjusted for, including daily mean temperature, temporal trend, public holidays, day of week, and relative humidity. There was a significant decrease in non-accident, cardiovascular and respiratory hospital admissions during the air pollution control period in Guangzhou. Controlling for potential confounding factors in the multivariate models did not significantly alter these findings. Similar effects were observed in the population for different disease categories (Cardiovascular conditions: Dysrhythmias, Dysrhythmias, Ischemic Heart Disease (IHD), and Peripheral and Cerebrovascular disease (PCD); Respiratory conditions: Pneumonia and COPD), in both sexes, and in all age groups. The daily time series of each measured air pollutant during the Asian Games period and baseline years are provided in Fig 2. It appears that daily NO_2_ concentrations significantly decreased during the Asian Games period (*P* = 0.027), and PM_10_ and NO_2_ experienced smaller daily variations during baseline years.

**Table 2:** Univariate and adjusted relative risk (RR) of hospital admissions during the 2010 Asian Games compared with the baseline period.

Hospital admission	Crude		Adjusted ^a^	
	RR(95%CI)	*P* value	RR(95%CI)	*P* value
**Causes of Hospital admission**				
**Non-accident**	0.827(0.805,0.851)	< .0001	0.810(0.788,0.833)	< .0001
**Cardiovascular**	0.789(0.720,0.865)	< .0001	0.766(0.699,0.840)	< .0001
**Respiratory**	0.827(0.773,0.884)	< .0001	0.812(0.759,0.868)	< .0001
**Cardiovascular**				
**Dysrhythmias**	0.562(0.385,0.822)	0.0031	0.549(0.375,0.805)	0.0022
**IHD**	0.750(0.636,0.885)	0.0007	0.746(0.633,0.881)	0.0006
**PCD**	0.781(0.645,0.945)	0.0113	0.777(0.642,0.941)	0.0102
**Respiratory**				
**Pneumonia**	0.614(0.544,0.692)	< .0001	0.607(0.538,0.684)	< .0001
**URI**	1.067(0.741,1.536)	0.7265	1.036(0.718,1.493)	0.8517
**Asthma**	0.883(0.679,1.147)	0.3515	0.827(0.635,1.077)	0.1602
**COPD**	0.837(0.744,0.942)	0.0032	0.818(0.727,0.921)	0.0009
**Sex**				
**Male**	0.827(0.795,0.861)	< .0001	0.811(0.779,0.844)	< .0001
**Female**	0.828(0.796,0.862)	< .0001	0.810(0.778,0.843)	< .0001
**Age**				
**2–18**	0.833(0.738,0.941)	0.0033	0.834(0.738,0.942)	0.0036
**19–64**	0.827(0.798,0.857)	< .0001	0.802(0.774,0.832)	< .0001
**65-**	0.845(0.802,0.890)	< .0001	0.831(0.788,0.875)	< .0001

^a^ Time-series poisson regression model with adjustment of day of week, public holidays, temporal trend, daily mean temperature, and relative humidity

Abbreviations: CI, confidence interval; PCD, peripheral and cerebrovascular disease; IHD, ischemic Heart Disease; URI, upper Respiratory Infection.

**Fig 3:**
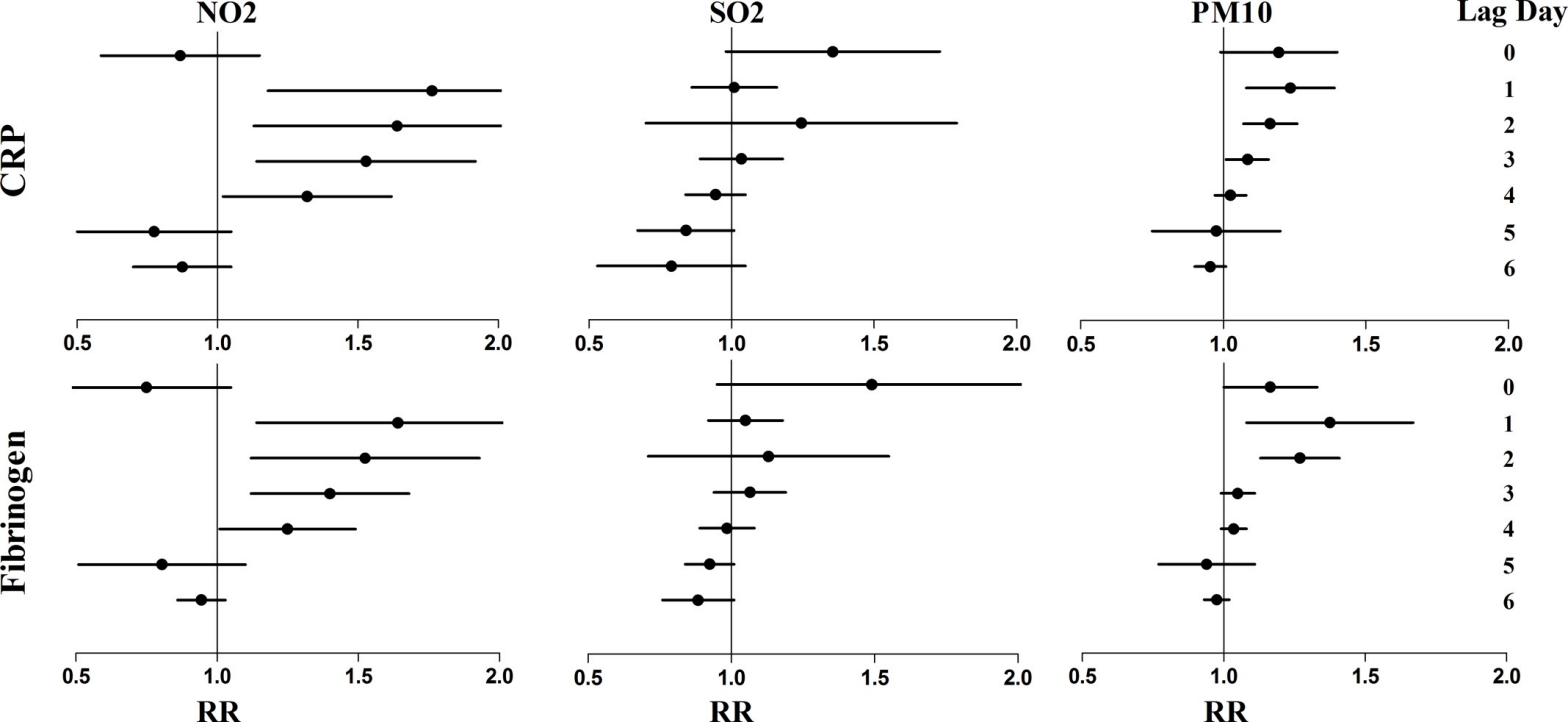
RRs and 95% CI estimates for the changes in biomarkers indicating systemic inflammation for CRP and fibrinogen associated with each 10 μg/m^3^ change in pollutant levels, by a 24-hour lag period. The lag time represents hours before a clinic visit. Zero lag represents 0 to 23 hours; lag 1, 24 to 47 hours; lag 2, 48 to 71 hours; lag 3, 72 to 95 hours; lag 4, 96 to 119 hours; lag 5, 120 to 143 hours; and lag 6, 144 to 167 hours.

Table 3 provides the measurements of 24-hour mean pollutant concentrations, relative humidity, and temperature by period. During the Asian Games period, the daily mean NO_2_ was 42.63 μg/m^3^, which was a 16.95% decrease compared to baseline conditions (Table 1). There were no significant decreases in other pollutants (PM_10_ and SO_2_) and meteorological conditions. We demonstrated statistically significant improved CRP levels by −20.4% (95%CI: -21.9% to -18.8%; *P* = 0.0010) from a pre-Asian game mean of 3.43 mg/L to a during game mean of 2.75 mg/L. Fibrinogen levels improved by -15.4% (95%CI: -18.1% to -12.6%; *P* = 0.0030) from 4.61 g/L to 3.91 g/L, using 2-sided tests performed at the 0.0083 level (Table 4). In the post-game period, when pollutant levels increased, most measurements approximated pre-game levels. CRP and fibrinogen levels both worsened significantly compared to the Asian Games period. We identified significant CRP increases that were related to increases in NO_2_ at lag 1–3 and PM_10_ at lag 1–2. Similar effects were also seen for fibrinogen (Fig 3).

**Table 3:** Distributions of 24-hour mean concentrations of pollutants, temperature, and relative humidity by period.

Pollutant and	Mean (SD)				Change % ^b^		Median (Range)			
Period ^a^	Entire	Before	During	After	Before to During	During to After	Entire	Before	During	After
	n = 153	n = 51	n = 51	n = 51			n = 153	n = 51	n = 51	n = 51
**NO**_**2**_, μ**g/m**^**3**^	45.1 (21.8)	48.3 (30.5)	42.6 (14.3)	44.5 (16.9)	-12.1	4.8	41(15–128)	36 (17–128)	41 (15–81)	43 (22–99)
**SO**_**2**_, μ**g/m**^**3**^	28.8 (15.2)	31.5 (18.1)	30.6 (15.1)	24.4 (10.7)	-3	-20.2	26 (4–73)	29 (4–73)	28 (4–63)	22 (5–49)
**PM**_**10**_, μ**g/m**^**3**^	64.3 (22.4)	63.9 (31.5)	62.6 (14.4)	66.4 (17.7)	-2.1	6.3	66 (9–135)	59 (9–135)	65 (17–83)	67 (20–105)
**Temperature,**°**C**	18.8 (4.5)	17.6 (5.1)	19.4 (3.5)	19.4 (4.6)	9.6	0	20 (6–28)	17 (9–28)	20 (6–23)	20 (11–28)
**Relative humidity, %**	60.0 (14.4)	64.6 (15.2)	57.7 (12.1)	57.8 (14.9)	-10.4	0	60 (23–91)	67 (31–91)	58 (36–89)	60 (23–89)

^*a*^ Before the Asian Games: November 1-December 21, 2009; during: November 1-December 21, 2010; after: November 1- December 21, 2011.

**Table 4:** Biomarker concentrations by period and between-period change in participant-specific biomarker concentrations in COPD patients.

	Asian Games Period ^a^ (No. of COPD = 36)			Between-Period Percentage Change			
Biomarker, Units	Before	During	After	Before to During		During to After	
	Mean(95%CI)	Mean(95%CI)	Mean(95%CI)	change% (95%CI)	*P* value ^b^	change%(95%CI)	*P* value ^b^
**CRP, mg/L**	3.43 (2.76, 4.10)	2.75 (2.20, 3.29)	3.21 (2.56, 3.87)	-20.4 (-21.9, -18.8)	**0.0010**	16.5 (14.0, 19.0)	**0.0020**
**Fibrinogen, g/L**	4.61 (3.92, 5.27)	3.91 (3.28, 4.54)	4.65 (3.97, 5.33)	-15.4 (-18.1, -12.6)	**0.0030**	21.8 (16.3, 27.4)	**0.0019**

^*a*^ Before the Asian Games: November 1-December 21, 2009; during: November 1-December 21, 2010; after: November 1- December 21, 2011

^b^ Significance is established if *P* value < 0.0083, the individual significance level needed to maintain a family-wise Type I error rate of 0.05

In Bold is *P* value < 0.0083.

Description of baseline characteristics for the cohort of 36 COPD patients have been showed in S1 Table. There were no significant differences between different periods (during the 2010 Asian Games compared with the baseline period). We compared the daily hospital admissions during the Asian Games period and the baseline years in the one control city, Xiangyang, Hubei Province (the control city detailed in S2 Table). There were no significant differences in hospital admissions for non-accidental, respiratory, and cardiovascular diseases. We also compared the hospital admissions for non-respiratory and cardiovascular diseases (Digestive system: K00-K99; Urogenital system: N00-N99) in the Asian Games period with the baseline years. These diseases were not etiologically related to air pollutants. We found the hospital admissions from non-respiratory as well as cardiovascular diseases did not significantly differ (the control diseases detailed in S3 Table). We further compared the daily hospital admissions and air pollutant concentration in non-Asian Games months (Jan-Oct, 2010) in the Games year (2010) and baseline years (2004–2009 and 2011–2013). There were no significant differences in hospital admissions for non-accidental, respiratory, and cardiovascular diseases (see the control city detailed in S4 Table in supplementary file). Throughout the study periods, PM_10_, NO_2,_ and SO_2_ were highly correlated (*r* > 0.69); the three pollutants were moderately correlated with mean temperature (S5 Table).

## Discussion

This study examined the impact of air quality changes on hospital admissions and blood biomarkers during the Asian Games in 2010 in Guangzhou, China. We concluded that reductions in urban ambient pollutants during the 2010 Asian Games was followed by a drop in hospital admissions for non-accidental, respiratory, and cardiovascular diseases. Moreover, there was a significant relationship between NO_2_ and PM_10_ and acute systemic inflammation (CRP and fibrinogen biomarkers) in COPD patients. This outcomes persisted, even when we adjusted for potential confounders.

During the implementation of the significant pollution control measures for the Asian games, we found moderate to large reductions in PM_10_ and NO_2_ concentrations, with the exception of SO_2_. In the post-Asian games period, once the pollution control measures were relaxed, the concentrations of these pollutants increased from the during-Asian games levels. Similar changes in pollution levels had also been previously reported for the Beijing Olympics [16, 17]. Corresponding beneficial public health effects have been reported to include reduced childhood hospital admissions for respiratory disease [18], reductions in respiratory and cardiovascular death rates [6], reductions in preterm deliveries [19], and reduced pulmonary inflammation in children [20]. Consistent with previous studies, we also found that lower air pollutant concentrations could reduce hospital admissions (total, respiratory, and cardiovascular diagnoses). A significant number of hospital admissions may be prevented by restricting transportation and controlling emissions from this study. This was consistent with a number of previous findings, although the major air pollutants accounting for the health benefits may differ [6, 21]. In addition, no differences by all ages and sex groups were observed in the present study.

Evidence linking ambient air pollutants to cardiovascular and respiratory health has been accumulating over the past decades. Studies reporting time-series analysis and cohort studies have demonstrated both short- and long-term impacts of air pollution on human health effects [22–26]. In a previous study, we found positive associations between hospital admissions for non-accidental, cardiovascular, and respiratory symptoms, and ambient exposures. In both single- and multi-pollutant models, NO_2_ is considered to be the unique pollutant with the greatest risk of hospital admissions for non-accidental and respiratory disease, especially for patients with COPD [9]. In this study, we further found that there were health benefits (reductions in hospital admissions for both cardiovascular and respiratory symptoms) associated with the air pollution interventions. These were stratified by categories of disease, with significant differences in hospital admissions being observed for IHD, and PCD of Cardiovascular diseases and in Pneumonia and COPD of respiratory diseases, during the 2010 Asian Games period compared with the baseline period. However, these previous studies did not identify the underlying reasons associated with the health benefits linked with the pollution interventions. As such, in the second part of the study, we further examined the potential causes between COPD and air pollutants.

Air pollutants may induce a low-grade systemic inflammatory state, possibly creating some mechanistic explorations[27]. The potential effects of air pollution on respiratory diseases involve the direct effect of air pollutants on the lung, and/or indirect effects that mediated by oxidative stress and pulmonary inflammation. These indirect effects may develop into a systemic inflammatory response [28]. Several panel researchers have found evidence to support these mechanisms in human subjects [29–31]. However, we identified only one other study that explored the impact of air pollution exposure on systemic inflammation markers in COPD patients [32]. Other studies have mainly relied either on healthy volunteers or cardiovascular disease patients [33–35]. Based on this study, the one air pollutant of most concern for COPD patients is NO_2_; these results were similar to a previous study [9]. Given the differences with that study, this research provided a unique opportunity to conduct such a design study, in which air pollution exposure and inflammatory markers were tested at baseline levels (pre-Asian games), following a decrease in pollution concentrations (during-Asian games), and then after an expected return to baseline (post-Asian games). We observed significant improvements in CRP and fibrinogen levels when comparing the pre-Asian game and the during-Asian game period. In the post-Asian game period, when pollutant concentrations were elevated, CRP and fibrinogen were statistically significantly worsened compared to the Asian Games period.

Inflammation is a very important factor in the pathogenesis of respiratory diseases [36], including these brought about by air pollutants [37]. Evidence suggests that short-term exposure to air pollution could trigger a COPD exacerbation episode, and that long-term exposure to air pollution could contribute to further COPD development and progression [38–41]. Acute-phase proteins are considered to be crucial biomarkers of systemic inflammation [42]. We measured fibrinogen and CRP on 36 stable COPD patients of Haizhu District for a three-year cohort study. Serum CRP is a pivotal acute phase reactant, with significant pro-inflammatory properties. It is considered to be a clinical indicator of inflammation. CRP levels are often low in healthy individuals but can increase quickly in response to inflammatory stimuli and other infection [43, 44]. Both in vitro and in vivo animal studies have shown that CRP levels increase following air pollutant exposure; however, studies examining the association of CRP with air pollutants are inconsistent [45]. For example, we identified increases in CRP concentration were associated elevated air pollution. This aligns with earlier field studies: increased CRP have been found in healthy men in Germany during an air pollution episode [46], and in a Coronary Heart Disease (CHD) cases study [47], although the pollutant concentrations and the RR of a single pollutant differed. However, similar analyses did not identify any effects in Regina Rucker et al’s study [48]. Several observational epidemiology studies have found that increased levels of fibrinogen are related with cardiovascular outcomes or even mortality [49, 50]. We also identified an increase in fibrinogen at lag 1–3 and lag 1–2 for NO_2_ and PM_10_, respectively.

In addition, some of the lag-day outcomes shown in the figures are intriguing. The changes in CRP and fibrinogen associated with NO_2_ and PM_10_ levels increased in the early average lag 1–3 periods. This is consistent with their roles as an acute phase protein [51–54]. For most markers, a gradual increase in the estimated effects was found in the early lag 0–2 days; then, there was a gradual decrease to often negative effects in later lag days (e.g., elevated pollutants are correlated with decreased biomarker levels). These so-called negative “protective” effects may be the result of compensatory mechanisms, because of the elevated biomarker levels caused by pollutants during the early lags. The overall associations of pollutant-biomarker provides further mechanistic support that changes in biomarker through the Asian game periods were considered to be acute inflammatory responses to the air pollution changes.

Finally, this study did have some limitations. First, the study did not evaluate genetic backgrounds and cigarette smoking, both of which may have underlying influences on daily hospital admissions in the long term. However, the GAM model used did include self-control in the analyses. As such, the impacts of potential confounding factors on the results were largely controlled. Second, given the nature of the Asian Games, the relatively short intervention period and the limited number of air pollution monitoring sites makes it difficult to draw firm conclusions. Finally, unmeasured medical or environmental factors may have significantly influenced our findings.

## Conclusion

During the Guangzhou Asian games, diminished air pollution was associated with reduced hospital admissions (for non-accidental, cardiovascular, and respiratory diseases), as well as an acute reduction in CRP and fibrinogen associated with systemic inflammation in COPD. Although the findings may not be clinically significant, this study provides mechanistic evidence to support the theory that ambient pollution might be a global adverse factor for health effects.

## Supporting information

S1 Fig Time series of daily hospital admission counts during the 2010 Asian Games period and during the baseline years in Guangzhou. (TIF)

S1 Table Demographic characteristics of COPD patients during the 2010 Asian Games compared with the baseline period. (DOCX)

S2 Table Distributions and relative risk (RR) of hospital admissions during the 2010 Asian Games compared with the baseline period in the one control city, Xiangyang. (DOCX)

S3 Table Distributions and relative risk (RR) of hospital admissions during the 2010 Asian Games compared with the baseline period in digestive system and urogenital system diseases in Guangzhou. (DOCX)

S4 Table Distributions and relative risk (RR) of the daily hospital admissions in non-Asian Games months (Jan-Oct, 2010) in the games year (2010) and baseline years (2004–2009 and 2011–2013) in Guang zhou. (DOCX)

S5 Table Spearman correlation coefficients for air pollutants, ambient temperature, and relative humidity, measured on a 24-hour basis. (DOCX)
