# Supplementary figures and images for: Impacts of event-specific air quality improvements on total hospital admissions and reduced systemic inflammation in COPD patients
Source: PLoS One. 2019 Mar 20;14(3):e0208687. doi: 10.1371/journal.pone.0208687 (PMC6426198; doi:10.1371/journal.pone.0208687)

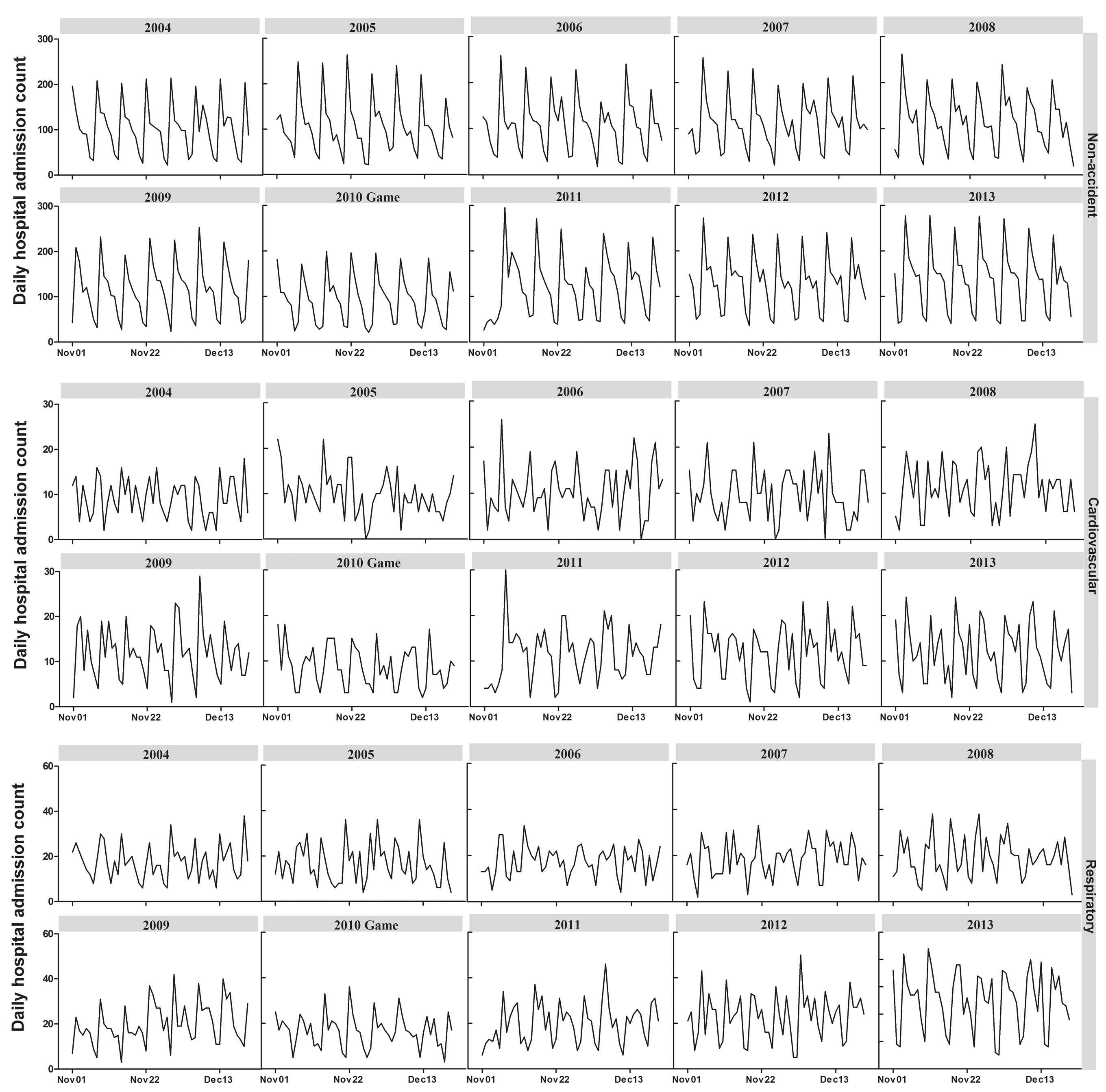

Supplement: S1 Fig — (TIF) [file pone.0208687.s001.tif]
