# Supplementary material for: Impacts of event-specific air quality improvements on total hospital admissions and reduced systemic inflammation in COPD patients
Source: PLoS One. 2019 Mar 20;14(3):e0208687. doi: 10.1371/journal.pone.0208687 (PMC6426198; doi:10.1371/journal.pone.0208687)
Supplement: S1 Table — (DOCX) [file pone.0208687.s002.docx]

**S1 Table. Demographic characteristics of COPD patients during the 2010 Asian Games compared with the baseline period.**

| **Variables** | **COPD** | | | ***P^a^*** |
| --- | --- | --- | --- | --- |
|  | **2009(n=41)** | **2010(n=38)** | **2011(n=36)** |  |
|  | n (%) | n (%) | n (%) |  |
| **Age** | 71.4±5.2 | 72.4±5.2 | 73.4±5.2 | >0.05 |
| **Sex** |  |  |  |  |
| **Male** | 41 (100) | 38 (100) | 36 (100) |  |
| **Female** | 0 | 0 | 0 |  |
| **Smoking status** |  |  |  |  |
| **Yes** | 0 | 0 | 0 |  |
| **No** | 41 (100) | 38 (100) | 36 (100) |  |
| **Kitchen fan usage** | 41 (100) | 38 (100) | 36 (100) |  |
| **Education** |  |  |  |  |
| **Primary or below** | 0 | 0 |  |  |
| **Junior or senior** | 7 (17.1) | 5 (13.2) | 4 (11.1) |  |
| **University or above** | 34 (82.9) | 33 (86.8) | 32 (88.9) |  |
| **Indoor air ventilation** |  |  |  |  |
| **Good** | 38 (92.7) | 35 (92.1) | 34 (94.4) |  |
| **Medium** | 3 (7.3) | 3 (7.9) | 2 (5.6) |  |
| **Bad** | 0 | 0 | 0 |  |
| **Cooking** |  |  |  |  |
| **Often** | 0 | 0 | 0 |  |
| **Sometimes** | 5 (12.2) | 4 (10.5) | 4 (11.1) |  |
| **Never** | 36 (87.8) | 34 (89.5) | 32 (88.9) |  |
| **GOLD** |  |  |  |  |
| **I** | 0 | 0 | 0 |  |
| **II** | 0 | 0 | 0 |  |
| **III** | 24 (58.5) | 21 (55.3) | 18 (50) |  |
| **IV** | 17 (41.5) | 17 (44.7) | 18 (50) |  |
| **Occupational exposures** | 0 | 0 | 0 |  |
| **Stable COPD** | 41 (100) | 38 (100) | 36 (100) |  |
| **Other respiratory diseases** | 0 | 0 | 0 |  |
| **Cancer** | 0 | 0 | 0 |  |
| **FEV1%, predicted** | 23.6±4.5 | 23.1±4.4 | 22.9±4.3 | >0.05 |
| **FEV1/FVC,%** | 34.8±8.4 | 33.6±8.8 | 33.3±8.5 | >0.05 |

*^a^* by repeated measures analysis of variance.
