# Supplementary material for: Impacts of event-specific air quality improvements on total hospital admissions and reduced systemic inflammation in COPD patients
Source: PLoS One. 2019 Mar 20;14(3):e0208687. doi: 10.1371/journal.pone.0208687 (PMC6426198; doi:10.1371/journal.pone.0208687)
Supplement: S2 Table — (DOCX) [file pone.0208687.s003.docx]

**S2 Table. Distributions and Relative risk (RR) of hospital admissions during the 2010 Asian Games compared with the baseline period in the one control city, Xiangyang.**

| **Hospital admission** | **Mean(SD)** | |  | **Adjusted ^c^** | |
| --- | --- | --- | --- | --- | --- |
|  | **Baseline period ^a^** | **Game period ^b^** |  | **RR (95%CI)** | ***P* value** |
| **Non-accident** | 148.9 (82.1) | 150.1 (78.8) |  | 1.01 (0.99-1.03) | 0.17 |
| **Cardiovascular** | 9.2 (4.6) | 9.3 (5.3) |  | 1.02(0.98-1.05) | 0.32 |
| **Respiratory** | 20.9 (9.3) | 21.2 (7.7) |  | 1.01(0.97-1.06) | 0.62 |

^a^ Baseline period represents November 1, to December 21 from 2008 to 2013, except 2010;

^b^ Games period represents November 1, to December 21 in 2010;

^c^ Time-series Poisson regression model with adjustment of day of week, public holidays, temporal trend, daily mean temperature, and relative humidity.
