# Supplementary material for: Impacts of event-specific air quality improvements on total hospital admissions and reduced systemic inflammation in COPD patients
Source: PLoS One. 2019 Mar 20;14(3):e0208687. doi: 10.1371/journal.pone.0208687 (PMC6426198; doi:10.1371/journal.pone.0208687)
Supplement: S3 Table — (DOCX) [file pone.0208687.s004.docx]

**S3 Table. Distributions and Relative risk (RR) of hospital admissions during the 2010 Asian Games compared with the baseline period in Digestive system and Urogenital system Diseases in Guangzhou.**

| **Hospital admission** | **Mean(SD)** | |  | **Adjusted ^c^** | |
| --- | --- | --- | --- | --- | --- |
|  | **Baseline period ^a^** | **Game period ^b^** |  | **RR (95%CI)** | ***P* value** |
| **Digestive system** | 6.8 (4.8) | 6.8 (4.5) |  | 0.98 (0.88-1.10) | 0.82 |
| **Urogenital system** | 13.0 (10.0) | 13.8 (10.2) |  | 0.95 (0.88-1.03) | 0.18 |

^a^ Baseline period represents November 1, to December 21 from 2004 to 2013, except 2010;

^b^ Games period represents November 1, to December 21 in 2010;

^c^ Time-series Poisson regression model with adjustment of day of week, public holidays, temporal trend, daily mean temperature, and relative humidity.
