# Supplementary material for: Impacts of event-specific air quality improvements on total hospital admissions and reduced systemic inflammation in COPD patients
Source: PLoS One. 2019 Mar 20;14(3):e0208687. doi: 10.1371/journal.pone.0208687 (PMC6426198; doi:10.1371/journal.pone.0208687)
Supplement: S4 Table — (DOCX) [file pone.0208687.s005.docx]

**S4 Table.** **Distributions and Relative risk (RR) of the daily hospital admissions in non-Asian Games months (Jan-Oct, 2010) in the Games year (2010) and baseline years (2004-2009 and 2011-2013) in Guang zhou.**

| **Hospital admission** | **Mean(SD)** | |  | **Adjusted ^c^** | |
| --- | --- | --- | --- | --- | --- |
|  | **Baseline period ^a^** | **Game period ^b^** |  | **RR (95%CI)** | ***P* value** |
| **Non-accident** | 101(58.1) | 102(65.9) |  | 1.02(0.98-1.05) | 0.23 |
| **Cardiovascular** | 8.9 (5.7) | 9.0 (5.1) |  | 0.99 (0.95-1.03) | 0.64 |
| **Respiratory** | 16.4 (8.1) | 17.7 (8.2) |  | 1.04 (0.99-1.08) | 0.08 |

^a^ Baseline period represents January 1, to October 31 from 2004 to 2013, except 2010;

^b^ Games period represents January 1, to October 31 in 2010;

^c^ Time-series Poisson regression model with adjustment of day of week, public holidays, temporal trend, daily mean temperature, and relative humidity.
