# Supplementary material for: Impacts of event-specific air quality improvements on total hospital admissions and reduced systemic inflammation in COPD patients
Source: PLoS One. 2019 Mar 20;14(3):e0208687. doi: 10.1371/journal.pone.0208687 (PMC6426198; doi:10.1371/journal.pone.0208687)
Supplement: S5 Table — (DOCX) [file pone.0208687.s006.docx]

**S5 Table. Spearman Correlation Coefficients for Air Pollutants, Ambient Temperature, and Relative Humidity, Measured on a 24-Hour Basis.**

| **Pollutants** | **NO_2_** | **SO_2_** | **PM_10_** | **Temperature** | **Relative** |
| --- | --- | --- | --- | --- | --- |
|  |  |  |  |  | **humidity** |
| **No. of 24-h periods** | 153 | 153 | 153 | 153 | 153 |
| **NO_2_** | 1 |  |  |  |  |
| **SO_2_** | 0.69 ^a^ | 1 |  |  |  |
| **PM_10_** | 0.83 ^a^ | 0.77 ^a^ | 1 |  |  |
| **Temperature** | 0.42 ^a^ | 0.31 ^a^ | 0.38 ^a^ | 1 |  |
| **Relative humidity** | 0.05 | 0.03 | 0.01 | 0.26 ^a^ | 1 |

*^a^ P* <0.01.
